# Effect of silver diamine fluoride and nanosilver on salivary bacterial counts in children with early childhood caries: a randomized controlled clinical trial

**DOI:** 10.1186/s12903-025-06325-3

**Published:** 2025-06-09

**Authors:** Nour Ammar, Magda M. El-Tekeya, Dalia M. Talat, Sara Essa, Marwa M. Essawy, Jan Kühnisch, Hams Hamed, Nouran Nabil, Samar El Achy, Maha El Tantawi

**Affiliations:** 1 Department of Pediatric Dentistry and Dental Public Health, Faculty of Dentistry, Alexandria University, Alexandria, 21521 Egypt; 2 Department of Medical Microbiology and Immunology, Faculty of Medicine, Alexandria University, Alexandria, 21521 Egypt; 3 Department of Oral Pathology, Faculty of Dentistry, Alexandria University, Alexandria, 21521 Egypt; 4 Center of Excellence for Research in Regenerative Medicine and Applications (CERRMA), Faculty of Medicine, Alexandria University, Alexandria, 21521 Egypt; 5 Department of Conservative Dentistry and Periodontology, University Hospital, Ludwig-Maximilian University of Munich, 80336 Munich, Germany; 6 Department of Pathology, Faculty of Medicine, Alexandria University, Alexandria, 21521 Egypt

**Keywords:** Pediatric dentistry, Dental caries, Nano silver, Nanoparticles, Saliva, Streptococci, Primary teeth, Early childhood caries

## Abstract

**Background:**

Silver diamine fluoride (SDF) is indicated for the management of early childhood caries (ECC). Similarly, nanosilver fluoride (NSF) is effective against caries. However, there are limited comparisons between both agents, especially regarding their antibacterial effect. This randomized controlled clinical trial aimed to compare the effects of SDF and NSF on salivary bacterial counts in children with ECC.

**Methods:**

Fifty 4-6-year-olds presenting with active dentin caries (ICDAS code 5) in primary teeth were randomly allocated to two groups. *Streptococcus mutans (S. mutans)* and *Lactobacilli* in unstimulated saliva were cultivated on differential media and counted as colony-forming units. Followed by the application of either 38% SDF or NSF. Saliva samples were recollected after one month. The legal guardians completed a detailed questionnaire assessing their child’s dental hygiene habits, dental pain experience, and socioeconomic background. Multivariable binary logistic regression was used to assess the effects of both agents on bacterial counts while accounting for confounders.

**Results:**

The mean age of participants was 4.8 ± 0.8 years, with the majority (96%, *N* = 48) presenting with severe ECC. There were no statistically significant differences between the two groups regarding age, sex, dmft score, socioeconomic background, dental hygiene habits, or dental experience. After one month, within-group analysis showed a significant reduction in *S. mutans* only in the NSF group (*p* = 0.002) and significant decreases in *Lactobacilli* counts in both SDF and NSF groups (*p* < 0.05). However, between-group comparisons revealed no significant differences in the reduction of *S. mutans* (1.4% and 6.0%, respectively, *p* = 0.192) or *Lactobacilli* counts (6.0% and 6.0%, respectively, *p* = 0.754). Regression analyses revealed non-significant odds of reduced bacterial counts after NSF application compared to SDF.

**Conclusion:**

After one application, children with ECC showed significant decrease in salivary bacteria, with no difference between the two agents regarding their antibacterial effect. NSF can serve as a viable option in ECC management in that it provides comparable antibacterial effects to 38% SDF without inducing tooth discoloration. This trial was prospectively registered on the clinicaltrials.gov registry with ID: NCT05221749 on 03/02/2022.

## Introduction

Early childhood caries (ECC) is a prevalent global health challenge, particularly in low- to lower-middle-income countries and incurs substantial economic burdens [1]. This problem is complicated by the inaccessibility of dental care to children in underprivileged regions [1] and the intertwined behavioral, social, and financial difficulties facing children in need of dental treatment [2]. Consequently, minimally invasive dentistry, particularly the use of silver diamine fluoride (SDF), has become popular for arresting caries and addressing extensive treatment needs over the last decade. It is especially useful since it does not require mechanical caries removal, is easily administered, and inexpensive [3].

Early childhood caries has a multifactorial etiology. This biofilm-mediated disease occurs in the presence of the cariogenic bacteria *Streptococcus mutans* (*S. mutans*), which metabolize glucose into lactic acid [4]. The acidogenic and aciduric effects of *S. mutans* are potentiated by synergism with *Lactobacilli* species colonizing the dental biofilm and causing further demineralization of the dental hard tissues [4]. SDF can potentially arrest caries and decrease the bacterial load through the release of silver ions, which are known to have antibacterial effects [3, 5]. SDF’s anti-caries and antibacterial effects may extend to other parts of the oral cavity via salivary diffusion [6, 7]. However, it causes tooth discoloration which can diminish patient acceptance and may reduce satisfaction with the appearance of treated teeth [3].

Silver nanoparticles (AgNPs) have potent antibacterial properties and are widely used in biomedical applications [5]. Clinical trials have shown that AgNPs can effectively arrest caries and decrease the bacterial load in carious lesions with an efficacy comparable to that of SDF, particularly when combined with fluoride to form nanosilver fluoride (NSF) [8–10]. Both SDF and NSF share the advantages of a simple and fast application suitable for managing children with ECC. Moreover, NSF has the advantage of not causing tooth discoloration [10].

SDF can decrease the salivary *S. mutans* counts when applied to carious teeth [6, 7, 11]. In contrast, there is a scarcity of clinical trials examining the effect of NSF on salivary *S. mutans* counts [12, 13]. Furthermore, little is known about the impact of NSF on salivary *Lactobacilli* in a clinical setting, and how its antibacterial effect compares to that of SDF. Comparing both agents’ antibacterial effects is important to assess the viability of NSF as a caries treatment. Especially, to overcome the discoloration associated with SDF use and provide another minimally invasive treatment option in areas with limited access to dental care. Thus, this study aimed to compare the antibacterial effect of SDF and NSF on salivary bacteria. The hypothesis was that there would be no differences in the salivary *S. mutans* and *Lactobacilli* counts after one month of SDF or NSF application in children with ECC manifested in dentin caries lesions.

## Methods

### Study design and ethical considerations

This parallel two-arm randomized controlled trial was compliant with the Helsinki Declaration as revised in 2013 and was prospectively registered on clinicaltrials.gov (#NCT05221749, registered on 03/02/2022). It took place at the department of Pediatric Dentistry and Dental Public Health, Faculty of Dentistry at Alexandria University, a university hospital which mainly serves disadvantaged populations since all dental treatments are provided free of charge. It serves over 14,000 children annually [14]. Ethical approval was granted by the ethics committee of the Faculty of Dentistry #0359–12/2021, and the trial was conducted per institutional and national ethical standards.

Detailed information was provided to the legal guardians about the study aims, interventions, and possible side effects. Written informed consent was obtained from the participants’ legal guardians. The children and their caregivers received age-appropriate oral hygiene instructions and were advised about the detrimental effects of frequent snacking and overconsumption of sugar-containing foods and drinks [2]. In addition, all recruited children were provided with the standard dental care needed beyond the scope of the study. No dental treatments — other than SDF or NSF application — were provided after the study began and until it ended.

### Sample size Estimation

After one month of SDF and NSF treatment, the mean log^10^ of *S. mutans* counts was 4.49 [7] and 0.002 [12], respectively. Using G*Power 3.1.9.7 [15] we followed Rosner’s method [16], with the alpha error set at 5%, power at 80%, and standard deviation (SD) at 4.49 [7]. This yielded a sample size of 17 children per group which was increased to 25 children per group to account for potential dropouts and processing errors, leading to a total sample size of 50 participants.

### Eligibility criteria

Participants had to fulfil the following inclusion criteria: 4- to 6-year-olds presenting with one or more dentin caries lesions in a vital deciduous tooth, which would constitute an ECC diagnosis [2]. Only active dentin caries corresponding to ICDAS code 5 were included [17]. We excluded children with special healthcare needs, children presenting with intraoral pathologies, or reporting the use of local or systemic antibacterial treatment within the last 14 days [11, 18]. Children who reported the use of topical fluoride products (i.e., mouthwashes or varnishes) within the last 14 days were also excluded. Additionally, teeth displaying signs of pulpitis or premature mobility were not considered.

### Dental examination

One examiner (five years of experience in pediatric dentistry) conducted the clinical examination for all participants. The investigator underwent a training and calibration phase for the use of the ICDAS by two experienced clinicians and showed high intra-examiner reliability (Kappa = 0.93 and 0.91 for the assessment of caries extension and activity, respectively). The clinical examination was carried out on a professional dental unit with appropriate illumination. Gross debris was removed with gauze and the teeth were dried with pressured air for 5 s. Using a plane surface mirror and a blunt-ended probe (CPI probe, Hu-Friedy, Chicago, IL, US), the examiner assessed the presence of ECC and severe ECC as defined by the [2] as well as recorded the caries experience as measured by the dmft index [19]. Caries activity was assessed following the ICDAS criteria where lesions were considered active when dentin felt soft/leathery on gentle probing and showed a dull/non-shiny surface [17]. Legal guardians filled out a WHO questionnaire [19] that gathered information about the family’s socioeconomic background and assessed the child’s dental hygiene habits —including brushing frequency, use of various dental cleaning tools (toothbrush, toothpaste, dental floss, miswak and/or other tools), type of toothpaste used, whether it was fluoridated and its concentration (if known). The questionnaire also inquired about the child’s dental experience regarding pain or problems over the past year and the frequency of dental visits in the past six months.

### Randomization, allocation, and blinding

The recruited participants were equally and randomly allocated to both groups (SDF or NSF) through a random number list generated by a computer. If a participant had multiple eligible teeth, all of these teeth were included in the study and received the same agent based on the participant’s random allocation. Only one agent was applied for each participant. The sequentially numbered opaque sealed envelopes method was used for allocation concealment. To limit bias, an assistant prepared the envelopes, which were handed to the clinician shortly before the application of the intervention. Given the characteristic bluish color of SDF and its ability to stain demineralized hard tissues, the operator could not be blinded to the intervention type, especially since NSF does not cause discernible tooth discoloration. The children, parents, microbiologist, and statistician were blinded to the intervention type.

### SDF and NSF agents

In the control group, 38% SDF (Advantage Arrest, Elevate Oral Care LLC, US) was used. This product is composed of silver ions, fluoride ions, and ammonia in an aqueous solution. In the test group, NSF was used. The NSF solution was prepared by the medical nanotechnology laboratory at the Center of Excellence for Research in Regenerative Medicine and its Applications at Alexandria University. The silver nanoparticles were synthesized according to the citrate reduction technique and coated with polyethylene glycol (PEG) to yield PEG-coated silver nanoparticles (PEG-AgNPs) [20, 21]. Characterization of the nano-suspension revealed PEG-AgNPs with 49 ± 5.9 nm average size and stabilized silver nanospheres with a surface charge of -35.9 ± 7.62 mV. Next, a concentration of 256 mg/mL PEG-AgNPs was achieved by adding 22,600 ppm of fluoride. The solution was stirred overnight in a lightproof container to ensure that the nanoparticles were uniformly dispersed.

The numerous biomedical applications and clinical trials emphasize the safety of AgNPs [5, 8, 10, 20, 22] particularly when stabilized with PEG coating [20]. We thoroughly assessed the cytotoxicity of the AgNPs before the RCT and verified that they showed a consistently high safety profile [21, 23]. The PEG-AgNPs showed plateau unreachable cytotoxic dose on both normal gingival fibroblast and cancerous cell lines with an additive selectivity index greatly tipped towards the cancerous cells, recording a half-maximal inhibitory concentration (IC50) of 3.9 × 10^17^ µM and 5.3 × 10^4^ µM, respectively. The findings confirmed the absence of safety concerns. Notably, the topical application of one drop (0.05 mL) of the solution is sufficient to treat 5 carious primary teeth. These results have been documented and published elsewhere [23, 24].

### Application of the agents

A standardized procedure was used to apply the SDF and NSF solutions. To enhance consistency, the same investigator applied the agents to all participants. After removing gross debris and isolating the selected tooth with cotton rolls and a saliva ejector, the cavity was air-dried for 5 s using oil-free compressed air. No carious tissue was removed. One drop of either agent was dispensed into a disposable plastic dappen dish and applied to the carious lesion using a microbrush (regular size, Microbrush International, Germany). If the patient had more than one eligible tooth included in the study, the agent was applied sequentially in a quadrant-wise fashion. After 24 h, the participants were called to record any adverse effects.

### Saliva sampling and analysis (outcome assessment)

The participants were instructed to refrain from eating for two hours prior to the appointment, and all appointments were scheduled in the morning. Saliva samples were collected twice, once immediately before applying the agents and then one month later. Participants were requested to tilt their heads forward and drool into a sterilized collection cup until one mL of unstimulated saliva was collected [6, 7]. The collection cups were tightly capped, transported to the Department of Medical Microbiology, and processed within an hour of saliva collection by an experienced microbiologist. The samples were vortexed for 30 s (ZX3 Advanced Vortex Mixer, VELP Scientifica Srl., Italy) and serially diluted tenfold with sterile saline. To cultivate *S. mutans*, Mitis Salivarius culture plates (BD Difco Mitis Salivarius Agar, Difco Laboratories Inc., New Jersey, US) were freshly prepared, to which ten mL of the prediluted samples were added. The plates were anaerobically incubated in a 10% CO_2_ environment (redLINE incubator model RI 115-U, Binder GmbH, Tuttlingen, Germany) for 72 h [6, 7]. Similarly, Rogosa agar plates (Rogosa SL Agar, HiMedia Laboratories, Mumbai, India) were prepared, inoculated, and aerobically incubated at 37 °C for 48 h to cultivate *Lactobacilli*. The microbiologist counted and identified the colonies on all plates. Colonies were identified and confirmed using film morphology, catalase test, and bile test. *S. mutans* were characterized as gram-positive cocci, displaying a negative catalase test, and a negative bile test. *Lactobacilli* were characterized as white mucoid colonies of long gram-positive non-spore-forming rods. Colony forming units/mL (CFU/mL) were counted using the equation:

$$\:\text{C}\text{F}\text{U}/\text{m}\text{L}=\frac{\text{n}\text{u}\text{m}\text{b}\text{e}\text{r}\:\text{o}\text{f}\:\text{c}\text{o}\text{l}\text{o}\text{n}\text{i}\text{e}\text{s}\:\times\:\text{d}\text{i}\text{l}\text{u}\text{t}\text{i}\text{o}\text{n}\:\text{f}\text{a}\text{c}\text{t}\text{o}\text{r}}{\text{v}\text{o}\text{l}\text{u}\text{m}\text{e}\:\left(\text{m}\text{L}\right)}$$

### Statistical analysis

Analysis was conducted using SPSS 28.0 (IBM Corp., Armonk, New York, USA). Normality was assessed using the Kolmogorov‒Smirnov test. Normally distributed variables were reported as mean and standard deviation (SD), while non-normally distributed variables were presented as median and interquartile range (IQR). The Mann‒Whitney U test was performed to compare the log CFU/mL between groups, and the effect size (Cohen’s d) was calculated for the between-group comparisons. Within the SDF and NSF groups, the differences in the bacterial log count between baseline and follow-up appointments were assessed using the Wilcoxon signed-rank test. An intention-to-treat analysis was used. The percentage change in bacterial count was calculated as:

$$\:\text{P}\text{e}\text{r}\text{c}\text{e}\text{n}\text{t}\:\text{c}\text{h}\text{a}\text{n}\text{g}\text{e}=\frac{\text{f}\text{o}\text{l}\text{l}\text{o}\text{w}\:\text{u}\text{p}\:\text{c}\text{o}\text{u}\text{n}\text{t}\:\--\:\text{b}\text{a}\text{s}\text{e}\text{l}\text{i}\text{n}\text{e}\:\text{c}\text{o}\text{u}\text{n}\text{t}}{\text{b}\text{a}\text{s}\text{e}\text{l}\text{i}\text{n}\text{e}\:\text{c}\text{o}\text{u}\text{n}\text{t}}\:\times\:\:100$$

Percent change was categorized as reduced (negative percent change) versus not reduced (zero and positive percent change) and this was the dependent variable in multivariable binary logistic regression. The independent variable was the type of agent. Data collected from the WHO questionnaire were included in the model as confounders: child’s age in years, child’s sex, toothbrushing frequency (at least once daily versus less), dental visits (at least once in the last six months versus less), and mother’s education (at least high school versus less). To optimize model validity and mitigate multicollinearity among confounders related to dental hygiene habits and experience, toothbrushing frequency was selected as the primary confounder representing oral hygiene practices. One model was developed for each type of bacteria. Significance was set at a p-value of 0.05. The adjusted odds ratios (aORs) and 95% confidence intervals (CI) were calculated. All tests performed were two-tailed.

## Results

**Fig. 1:**
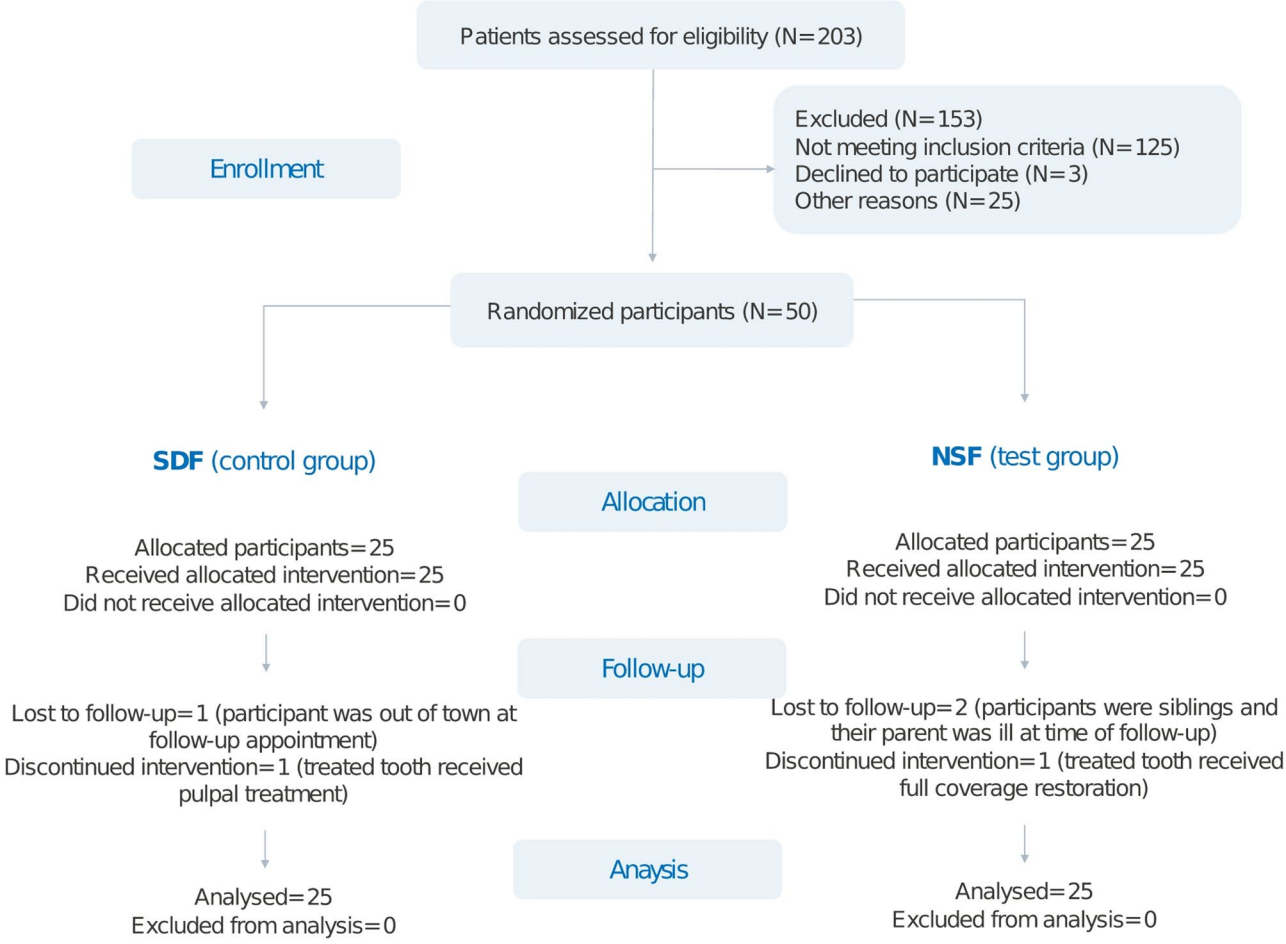
Study participants flow chart

The mean ± SD age of the 50 participants was 4.8 ± 0.8 years. The median (IQR) dmft scores for the SDF and NSF groups were 9.0 (3.7) and 9.8 (4.3), respectively. Two children presented with ECC, and 48 children suffered from severe ECC. There were no statistically significant differences between the two groups regarding age, sex, dmft score, dental hygiene habits, or dental experience. Most participants reported that their children brushed their teeth less than once daily and took their children to the dentist at least once within the last 6 months (Table 1). Five participants were lost to follow-up. Nonetheless, all recruited participants were accounted for by applying the intention-to-treat analysis according to their original group assignment (Fig. 1). None of the participants reported pain or soft tissue injury after the application of either intervention. No adverse effects were reported throughout the study duration.

**Table 1:** Sociodemographic profile and oral health behaviours of the study groups

		SDF 25 participants, 59 teeth	NSF 25 participants, 71 teeth	*p*-value
Age	Mean (±SD)	4.9 (±0.9)	4.6 (±0.6)	0.199
Sex	Male N (%)	12 (48.0%)	14 (56.0%)	0.571
	Female N (%)	13 (52.0%)	11 (44.0%)	
Decayed teeth (dt)	Mean (±SD)	7.2 (±3.0)	8.0 (±4.4)	0.785
Missing teeth (mt)		0.4 (±1.3)	0.1 (±0.3)	0.918
Filled teeth (ft)		1.5 (±1.4)	1.6 (±2.0)	0.826
dmft		9.1 (±3.7)	9.7 (±4.3)	0.585
Number of active cavities (ICDAS code 5)/patient	Mean (±SD)	3.6 (±1.9)	3.6 (±1.6)	1.000
Tooth type	Anterior N (%)	19 (32.2%)	29 (40.8%)	0.309
	Posterior N (%)	40 (67.8%)	42 (59.2%)	
Mother Education	Less than high school N (%)	12 (48.0%)	14 (56.0%)	0.577
	High school & higher N (%)	13 (52.0%)	11 (44.0%)	
Tooth brushing frequency	Less than once daily N (%)	14 (56.0%)	17 (68.0%)	0.382
	At least once daily N (%)	11 (44.0%)	8 (32.0%)	
Dental visits in last 6 months	At least once N (%)	19 (76.0%)	15 (60.0%)	0.225
	Less than once/never N (%)	6 (24.0%)	10 (40.0%)	

**Fig. 2:**
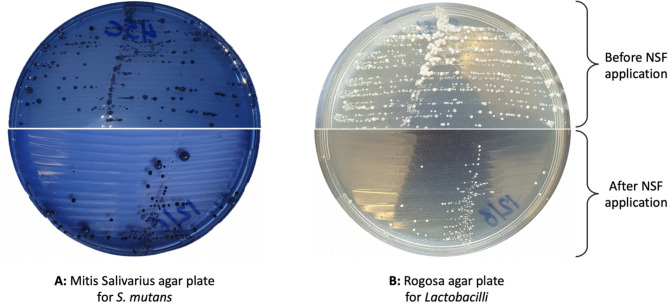
Agar plates of a saliva sample before (upper half) and after (bottom half) NSF application. **A**: Mitis Salivarius agar for the culture of *S. mutans*. **B**: Rogosa agar for the detection of *Lactobacilli*

At baseline, there were no significant differences in the median (IQR) log₁₀ *S. mutans* between the SDF and the NSF groups (4.9 (0.5) and 4.9 (0.3), *p* = 0.245) or the *Lactobacilli* levels (4.6 (0.7) and 4.7 (0.7), *p* = 0.745) (Fig. 2). After 1 month, the SDF and NSF groups showed reduction in *S. mutans* counts to 4.5 (0.5) and 4.5 (0.9) with no significant between-group difference in log counts (*p* = 0.783, Cohen’s d = 0.285) or percent reductions (1.4% (16.5) and 6.0% (20.0), *p* = 0.192, Cohen’s d = 0.530) (Table 2). The within group reduction in the SDF group was not significant (*p* = 0.270) but was significant in the NSF (*p* = 0.002). For *Lactobacilli*, after 1-month, the median (IQR) log counts in the SDF and NSF groups were 4.3 (0.7) and 4.0 (4.5), with no significant between group difference in log values (*p* = 0.337, Cohen’s d = 0.387), or percent reduction (6.0% (13.2) and 6.0% (60.0); *p* = 0.754, Cohen’s d = 0.019). There were significant reductions within the SDF and NSF groups (*p* < 0.001 and 0.007).

**Table 2:** Comparison of *S. mutans* and *Lactobacilli* counts between the study groups at baseline and after 1 month

			SDF 25 participants, 59 teeth	NSF 25 participants, 71 teeth	p-value
			Median (IQR)	Median (IQR)	
***S. mutans***	Baseline	Count	7.0 (7.0) × 10^4^	8.0 (5.0) × 10^4^	0.245
		Log10	4.9 (0.5)	4.9 (0.3)	
	1 month	Count	3.0 (3.4) × 10^4^	3.0 (3.8) × 10^4^	0.783
		Log10	4.5 (0.5)	4.5 (0.9)	
	% Reduction		1.4 (16.5)	6.0 (20.0)	0.192
	p-value		0.270	0.002*	
***Lactobacilli***	Baseline	Count	4.0 (8.0) × 10^4^	5.0 (7.0) × 10^4^	0.745
		Log10	4.6 (0.7)	4.7 (0.7)	
	1 month	Count	2.0 (2.4) × 10^4^	1.0 (3.0) × 10^4^	0.337
		Log10	4.3 (0.7)	4.0 (4.5)	
	% Reduction		6.0 (13.2)	6.0 (60.0)	0.754
	p-value		<0.001*	0.007*	

Table 3 shows the results from the regression model for factors affecting the presence of reduction in salivary bacterial counts after one month. Participants who received NSF were less likely to experience a decrease in *S. mutans* counts than those who received SDF (aOR = 0.85, 95% CI: 0.24–3.05), with no significant difference between groups (*p* = 0.801). Participants who received NSF were 1.15 times more likely to have reduced *Lactobacilli* counts than those with SDF (95% CI: 0.33–3.99), with no significant difference between groups (*p* = 0.822).

**Table 3:** Multivariable binary logistic regression assessing the effect of NSF versus SDF on the reduction of salivary *S. mutans* and *Lactobacilli*

	S. mutans	Lactobacilli
aOR	0.85	1.15
95% Cl	0.24–3.05	0.33–3.99
p-value	0.801	0.822
Log likelihood (-2LL)	59.73	61.13
% Correctly classified	74.0%	56.0%
Model’s p-value	0.263	0.756

aOR: adjusted odds ratio, CI: confidence interval

The models were adjusted for child’s age, sex, toothbrushing frequency, dental visits number, and mother’s education

## Discussion

This RCT compared the short-term antibacterial effects of SDF and NSF on salivary *S. mutans* and *Lactobacilli* in children suffering from ECC. *S. mutans* remains the most strongly associated bacterium with caries. Along with *Lactobacilli*, they are often cited prominent contributors to decay progression [4]. The results show that both agents led to a significant but minor reduction in bacterial counts after one month, with no difference in magnitude between groups. Therefore, the null hypothesis was rejected. The findings shed light on the antibacterial effect of these fluoride agents and provide information that can inform decision-making for minimally invasive management of ECC in children with advanced disease levels.

The results are in line with previous studies, although the magnitude of reduction recorded in the present study is lower. This may be partly attributed to the low baseline *S. mutans* count in the study participants (Table 2). Moreover, in comparable published studies, additional interventions were carried out simultaneously with agent application, which could account for the variations observed. In contrast to the present study where no caries excavation was done [6], excavated caries using atraumatic restorative technique (ART) then applied the SDF and reported a 40.2% decrease in *S. mutans* counts. On the other hand [7], reported a reduction in *S. mutans* counts of about 17% which may be related to their higher baseline *S. mutans* count. The two aforementioned studies recruited participants with mixed dentition with mild to moderate caries, while the present study focused on ECC presenting in extensive decay only. It is worth noting that a similar study documented a two-fold increase in *S. mutans* counts following three months of SDF application in children [11]. Only one study examined the effect of SDF on salivary *Lactobacilli* counts, reporting a decrease of 18.0% [11]. It is important to note that the investigators in that study enhanced the effects of SDF by applying fluoride varnish to the entire dentition during the same visit of SDF application. Consequently, the reported reduction cannot be exclusively attributed to SDF’s effect. Our study, thus, fills a knowledge gap by providing evidence about the impact on salivary *S. mutans* and *Lactobacilli* after one month of SDF application on dentin lesions in primary teeth.

Regarding NSF, Waikhom et al. 2022 [12] applied NSF varnish to the entire dentition of school-age children with no decay or with non-cavitated lesions and reported a significant decrease in *S. mutans* counts in saliva samples, some as high as 99%. The difference in the magnitude of reduction between their and our study can be attributed to the number of teeth included, caries level, in addition to nanoparticle size and delivery vehicle. Similarly, a 24-hour pilot study investigating the effect of NSF on tooth biofilm concluded that the dental biofilm of NSF-treated teeth showed a significant decrease in *S. mutans* counts after treatment [13]. To the best of our knowledge, we were unable to find clinical trials investigating the effect of NSF on salivary *Lactobacilli.* However, an in-vitro study demonstrated that *Lactobacilli* are highly susceptible to AgNPs [25]. The present study addresses this gap in the literature. Furthermore, it provides a direct comparison of the effects of SDF and NSF on salivary bacteria, thus also filling another knowledge gap.

Similar to prior research, the current study collected saliva samples to evaluate bacterial counts [6, 7, 26]. Site or tooth-specific biofilm sampling usually shows more remarkable changes than pooled samples (i.e., saliva) which reflect the collateral effects of an intervention in the oral cavity. There is no standardized regimen for oral microbiological analysis. The most widely used method is culturing using species-specific media in a dedicated microbiology laboratory [26], which was used in the present trial. Notably, collecting saliva samples from children is particularly advantageous since it requires less patient cooperation than tooth-specific samples.

NSF exerts its antibacterial effect primarily through the synergistic action of AgNPs and fluoride, with the nanoparticles playing a central role through their interactions with bacterial cell membranes. These nanoparticles penetrate the bacterial cell wall and induce direct and indirect lipid peroxidation damaging the cell membrane and disrupting DNA replication and repair ultimately leading to cell death [8, 27]. The antibacterial effects of fluoride are well-studied [8, 28]. It contributes to the overall antimicrobial activity and supports remineralization rendering NSF an effective and biocompatible agent against oral bacteria [9, 23, 29].

Among the strengths of the current study is the randomized controlled trial design conducted among preschool-aged children, which presents a challenge in behavioral management and clinical treatment. Additionally, multivariable regression was implemented to restrain the effects of confounders. The trial also addressed an area of scarce research [12, 13], and provided information that may reflect the situation in teeth other than those on which the agents were directly applied, or in the oral cavity in general.

Alternatively, the one-month follow-up period is a limitation. Other studies reported longer follow-up durations of up to three or six months [7, 9]. However, the antibacterial effects of topical interventions are not typically sustained over extended durations. Oral biofilms re-establish within days after disruption, with significant recolonization typically occurring within 3–4 weeks, making a one-month interval appropriate for capturing the early effects of antimicrobial agents [30, 31]. This duration provides a practical and scientifically sound timeframe to evaluate the primary efficacy of the interventions, while longer-term effects could be explored in future research. Previous studies with SDF or NSF assessed the change in bacterial levels after one day [7, 13], three days [32], 2 weeks [33], and 21 days [33] with many studies using a follow-up period of one-month [6, 7, 12, 18].

Although operator blinding was not possible due to visible tooth staining, several measures were taken to minimize this potential source of bias. Both the microbiologist and the statistician were fully blinded to group allocation, ensuring objective outcome assessment and data interpretation. Additionally, all treatment procedures were strictly standardized, and the primary outcomes were based on quantitative microbial counts rather than subjective measures. These measures helped mitigate the risk of bias associated with the unblinded operator.

The present trial included a population of children suffering from severe ECC with an average dmft of 9.3 (Table 1). The study setting in a public hospital serving patients with limited oral health literacy is a possible explanation for the fact that both interventions showed a marginal effect on bacterial counts (Table 2). Furthermore, the adherence of the participating children to daily oral hygiene instructions was not monitored. A factor to possibly consider in future studies is assessing the impact of toothbrushing on reducing the bacterial load and teasing out its effect from that of the topical fluoride agents in children with low brushing frequency, similar to those included in the present study.

Future studies with different microbiological analysis techniques could provide in-depth insights into the effect of the investigated antibacterial agents on oral bacteria. The trial addressed an area of scarce research [12, 13] and provided information about the effect of two minimally invasive anti-caries agents on salivary bacteria. This information may reflect the situation in teeth other than those on which the agents were directly applied suggesting a potential benefit to the whole oral cavity. The limited reduction of bacterial counts suggests that the antibacterial effect of both agents may be secondary to their remineralizing effect or their site-/tooth-specific antibacterial activity, as reported in our previous publication [24]. This aspect remains a subject for future investigations.

## Conclusions

After one month, children with ECC treated with SDF or NSF showed a minimal but significant decrease in salivary counts of S. mutans and Lactobacilli. There were no differences in the antibacterial effects of both agents. The results highlight the potential benefits of SDF and NSF in ECC management and address a knowledge gap. NSF can serve as a viable option in ECC management since it provides comparable antibacterial effects to 38% SDF without inducing tooth discoloration—an advantage for patient and parental acceptance. Further investigations are warranted to develop a comprehensive understanding of the advantages of using both agents for ECC management.

## Data Availability

The dataset used in this research is publicly available on synapse.org. Synapse ID: syn43185346, which can be found at https://www.synapse.org/#!Synapse:syn43185346/files/.

## Abbreviations

SDF: Silver diamine fluoride
ECC: Early childhood caries
NSF: Nanosilver fluoride
S. mutans: Streptococcus mutans
AgNPs: Silver nanoparticles
PEG: Polyethylene glycol
PEG-AgNPs: PEG-coated silver nanoparticles
IC50: Half-maximal inhibitory concentration
IQR: Interquartile range
aORs: Adjusted odds ratios
CI: Confidence intervals
